# Bioactivity profile of dissolved organic matter and its relation to molecular composition

**DOI:** 10.1007/s13659-023-00395-y

**Published:** 2023-09-18

**Authors:** Teresa S. Catalá, Linn G. Speidel, Arlette Wenzel-Storjohann, Thorsten Dittmar, Deniz Tasdemir

**Affiliations:** 1 Global Society Institute, Wälderhaus, Hamburg Germany; 2 Organization for Science, Education and Global Society gGmbH, Stuttgart, Germany; 3 ICBM-MPI Bridging Group for Marine Geochemistry, Institute for Chemistry and Biology of the Marine Environment (ICBM), University of Oldenburg, Oldenburg, Germany; 4 Geological Institute, Department of Earth Sciences, ETH Zurich, 8092 Zurich, Switzerland; 5 GEOMAR Centre for Marine Biotechnology, Research Unit Marine Natural Products Chemistry, GEOMAR Helmholtz Centre for Ocean Research Kiel, Am Kiel-Kanal 44, 24106 Kiel, Germany; 6 Helmholtz Institute for Functional Marine Biodiversity, University of Oldenburg, Oldenburg, Germany; 7 Kiel University, Christian-Albrechts-Platz 4, 24118 Kiel, Germany

**Keywords:** Dissolved organic matter, Antibacterial activity, Antifungal activity, Antioxidant activity, Molecular composition

## Abstract

Dissolved organic matter (DOM) occupies a huge and uncharted molecular space. Given its properties, DOM can be presented as a promising biotechnological resource. However, research into bioactivities of DOM is still in early stages. In this study, the biotechnological potential of terrestrial and marine DOM, its molecular composition and their relationships are investigated. Samples were screened for their in vitro antibacterial, antifungal, anticancer and antioxidant activities. Antibacterial activity was detected against *Staphylococcus* *aureus* in almost all DOM samples, with freshwater DOM showing the lowest IC_50_ values. Most samples also inhibited *Staphylococcus* *epidermidis*, and four DOM extracts showed up to fourfold higher potency than the reference drug. Antifungal activity was limited to only porewater DOM towards human dermatophyte *Trichophyton* *rubrum*. No significant in vitro anticancer activity was observed. Low antioxidant potential was exerted. The molecular characterization by FT-ICR MS allowed a broad compositional overview. Three main distinguished groups have been identified by PCoA analyses. Antibacterial activities are related to high aromaticity content and highly-unsaturated molecular formulae (O-poor). Antifungal effect is correlated with highly-unsaturated molecular formulae (O-rich). Antioxidant activity is positively related to the presence of double bonds and polyphenols. This study evidenced for the first time antibacterial and antifungal activity in DOM with potential applications in cosmeceutical, pharmaceutical and aquaculture industry. The lack of cytotoxicity and the almost unlimited presence of this organic material may open new avenues in future marine bioprospecting efforts.

**Graphical abstract:**

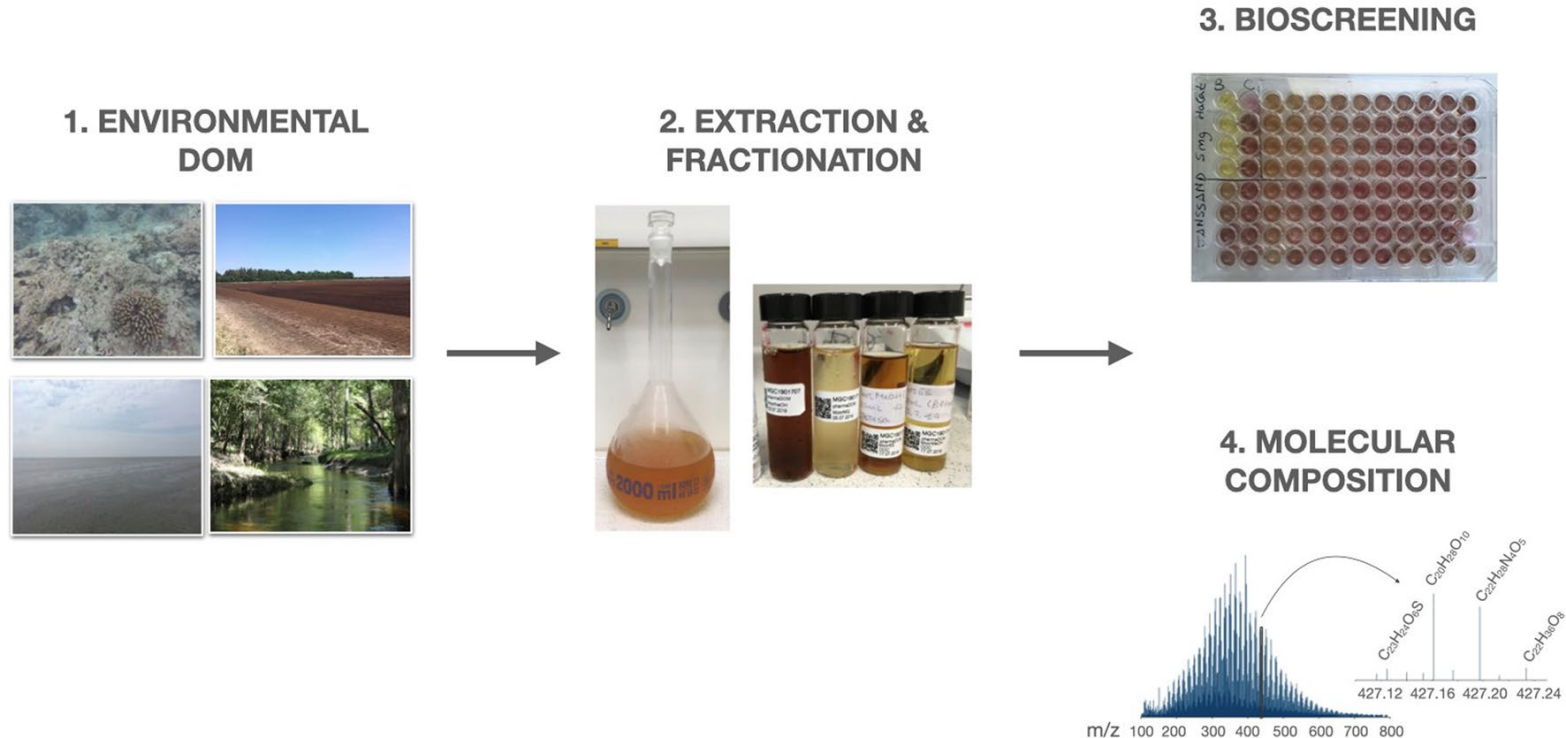

**Supplementary Information:**

The online version contains supplementary material available at 10.1007/s13659-023-00395-y.

## Introduction

Dissolved organic matter (DOM) refers to a complex mixture of organic compounds that are present in natural waters, such as rivers, lakes, and oceans [1]. It is a heterogeneous mixture composed of a wide range of organic molecules, including humic and fulvic acids, proteins, lipids, carbohydrates, and other compounds that are derived from living and decaying organisms [2]. It is one of the most significant sources of bioavailable organic carbon in aquatic ecosystems, therefore playing a critical role in many biogeochemical processes, such as nutrient cycling, carbon storage and transport [3, 4]. Most of the released DOM is quickly consumed by heterotrophs (heterotrophic bacteria, archaea and eukaryotic microorganisms) within hours to days [5, 6]. A minor fraction escapes microbial remineralization, decomposing slowly enough to persist for millennia, making most of the perceptible DOM pool [7] and giving rise to global carbon estimations of ca. 660 Pg (1 Pg C = 10^15^ g C) in the ocean [8] and up to 1.7 Pg C in freshwaters [9], at concentrations < 1 mg L^−1^ in seawater [10, 11] and 0.1–332 mg L^−1^ in freshwaters [12–14].

During microbial decomposition, DOM is molecularly diversified [15], giving rise to one of the most complex mixtures of Earth [16]. Although the causes of diversity are not well understood, it is known that enzymatic reactions are responsible of the formation and degradation of DOM [17]. Most DOM compounds have low molecular mass (< 1.000 Da) [18]. More than 20.000 molecular formulae have been identified with ultrahigh-resolution mass spectrometry [19], with 30 or more isomers per formula [16]. Therefore, the characterization of molecular structures of the possibly many millions of different organic compounds in DOM (below picomolar concentrations) has been a long-standing analytical challenge [20, 21]. Hence, the molecular structure and concentration of a minor fraction of the molecules held in DOM is known [7]. Amino acids or carbohydrates in the freshly-produced DOM category [22–24], lipophilic compounds [25] and microbial lipids [26] in aged DOM have been identified.

Nature is the most ancient pharmacy that provided cure for all kind of human ailments for centuries. Despite the short history of marine biodiscovery, the number of reported marine natural molecules exceed 35,000 [27] and the tremendous biodiversity found in the oceans is widely regarded as one of the most promising sources for discovery and development of new medicines. Current global marine pharmaceutical pipeline contains 17 marine drugs derived from marine macro- and microorganisms (and their semi-synthetic or synthetic derivatives) that have been approved for clinical use for treatment of cancer, pain, or obesity, while approx. 40 marine derived molecules are undergoing different stages of clinical trials worldwide [28]. Besides pharmaceuticals, marine organisms, their extracts, special fractions or purified metabolites find endless uses in other areas, such as food, cosmetics, agri- or aquaculture [29]. Despite the extensive studies on biogeochemistry and geographical ubiquity of DOM, this resource has rarely been considered as a potential bioresource in marine bioprospecting, consequently only a few studies have proved bioactivities, such as anti-atherosclerotic and anti-platelet aggregation effect, antiviral, and antioxidant activity of DOM from different aquatic sources [30–36]. Due to complex chemical composition of the DOM pool, all these studies performed sophisticated multivariate statistics and correlation analyses, but did not attempt fractionating or purifying the individual components of DOM extracts.

In this study, we aim at expanding knowledge on biotechnological potential of DOM and introduce it as a novel and untapped source of bioactive natural products relevant for the pharmaceutical, cosmeceutical and aquaculture industry. Given that this resource has been scarcely studied in any environment from this point of view, we investigated both terrestrial and marine DOM extracts (and polarity fractions thereof). The main aims of this study were (1) screening of bulk DOM extracts for their in vitro antibacterial, antifungal, anticancer and antioxidant activities, and (2) molecular characterization of DOM extracts with ultra-high resolution mass spectrometry (FT-ICR MS), to finally (3) draw relationships between DOM molecular composition and its bioactive potential.

## Results and discussion

### Bioactivity screening of DOM

The first tests were accomplished using DOM from different origins as well as some of its fractions with sufficient amounts. Samples were tested at an initial concentration of 200 µg/mL. The extracts that showed ≥ 50% activity at this initial testing were subjected to a serial dilution series to obtain their IC_50_ values (half maximal inhibitory activity). Unfortunately, several samples e.g., PW_50MeOH_, PW_100EA_, FW_100EA_ and DW_50MeOH_ samples provided insufficient amounts and could not be further tested (shown as n.t. in Table 1).

**Table 1:** Biological activity of the different DOM sources and their fractions. IC_50_ values are expressed in mg/mL

Sample code	Sa	Psa	Efm	Ef	Ecas	Lg	Vp	Ca	Cn	Tru	A-375	HCT-116	MB-231	Cac	Se	Tyro	DPPH	CAA* %
P_NaOH_	56.7 (±4.2)	>200	>200	>200	>200	>200	>200	>200	>200	>200	>200	>200	>200	>200	11.9 (± 1.2)	>200	n.t.	n.t.
P_MQ_	45.6 (± 3.9)	>200	>200	>200	>200	>200	>200	>200	>200	>200	>200	>200	>200	>200	4.0 (± 1.7)	>200	144.6 (± 1.7)	n.t.
P_MeOH_	19.2 (± 2.0)	>200	>200	>200	>200	>200	>200	>200	>200	>200	>200	>200	>200	>200	7.2 (± 0.3)	>200	61.7 (± 0.7)	53
P_EA_	56.6 (± 21.1)	>200	>200	>200	>200	>200	>200	>200	>200	>200	>200	>200	>200	>200	24.9 (± 3.6)	>200	> 200	n.t.
PW_bulk_	181.2 (± 9.4)	>200	>200	>200	>200	>200	>200	>200	>200	18.2 (± 0.4)	>200	>200	>200	>200	143.0 (± 6.6)	>200	> 200	-
DW_bulk_	20.1 (± 1.5)	>200	>200	>200	>200	>200	>200	>200	>200	>200	>200	>200	>200	>200	> 200	>200	> 200	28
FW_bulk_	7.8 (± 0.4)	>200	>200	>200	>200	>200	>200	>200	>200	>200	>200	>200	>200	>200	6.8 (± 1.3)	>200	195.0 (± 4.5)	–
PW_80MeOH_	35.1 (± 2.0)	>200	>200	>200	>200	>200	>200	>200	>200	>200	>200	>200	>200	>200	> 200	>200	140.5 (±4.0)	–
FW_50MeOH_	179.9 (± 4.1)	>200	>200	>200	>200	>200	>200	>200	>200	>200	>200	>200	>200	>200	> 100	>200	> 200	–
FW_80MeOH_	9.7 (± 3.8)	>200	>200	>200	>200	>200	>200	>200	>200	>200	>200	>200	>200	>200	2.2 (± 0.1)	>200	135.7 (±0.8)	–
DW_80MeOH_	>200	>200	>200	>200	>200	>200	>200	>200	>200	>200	>200	>200	>200	>200	> 200	>200	> 200	–
DW_100EA_	65.3 (± 0.8)	>200	>200	>200	36.3 (± 13.9)	>200	>200	>200	>200	>200	>200	>200	>200	>200	58.3 (± 1.4)	>200	> 200	51
Pos. control	0.7 (± 0.0)				0.9 (± 0.0)					0.1 (± 0.0)					8.0 (± 0.0)		4.1 (± 0.0)	88*%

Sa (*S. aureus*), Psa (*P. aeruginosa*), Efm (*E. faecium*), Ef (*E. faecalis*), Ecas (*Enterococcus casseliflavus*), Lg (*L. garvieae*), Vp (*V. parahaemolyticus*), Ca (*C. albicans*), Cn (*C. neoformans*), Tru (*T. rubrum*), A-375 (melanoma), HCT-116 (colon cancer), MB-231 (breast cancer), Cac (*C. acnes*), Se (*S. epidermidis*), Tyro (Tyrosinase inhibition assay), DPPH (cell-free antioxidant assay), CAA (cellular antioxidant assay). Positive controls: chloramphenicol (*S. aureus, S. epidermidis, V. parahaemolyticus, C. acnes*), polymyxin B (*P. aeruginosa*), ampicillin (*E. faecium*, *E. faecalis*, *E*. *casseliflavus, L. garvieae*), nystatin (*C. albicans*), amphotericin B (*C. neoformans*), clotrimazole (*T. rubrum*), doxorubicin (A-375, HCT-116, MB-231), kojic acid (Tyrosinase), ascorbic acid (DPPH), luteolin (CAA)

* % Inhibition, IC50 values were not determined due to low sample amounts, n.t.: not tested

#### Antimicrobial activity

In this study, a first evaluation of the DOM antibacterial potential has been conducted. Nine clinically relevant bacterial strains were tested. As shown in Table 1, significant antibacterial activity was detected in almost all original DOM bulks and fractions against the human pathogen *Staphylococcus* *aureus* that can cause small infections to life-threatening infections ane even sepsis. The most potent samples originated from freshwater, i.e., FW_bulk_ (IC_50_ value 7.8 µg/mL) and its fraction FW_80MeOH_ (IC_50_ value 9.7 µg/mL). The only inactive sample was the fraction DW_80MeOH_, while the remaining samples have shown IC_50_ values ranging from 20.1 to 181.2 µg/mL.

No activity was observed against the gram-negative human pathogen *Pseudomonas* *aeruginosa*, nor towards the gram-positive pathogens *Enterococcus* *faecium* or *E.* *faecalis* at 200 µg/mL concentration. DW_100EA_ was the only sample that inhibited the growth of *E.* *casseliflavus* (IC_50_ value 36.3 µg/mL). None of the samples proved active against *Lactococcus* *garvieae* or *Vibrio* *parahaemolyticus*, pathogenic bacteria that cause infections in native or aquacultured fish/shellfish and may be transmitted to human through contaminated seafood.

Human pathogenic yeasts, *Candida* *albicans* and *Cryptococcus* *neoformans* were fully insusceptible towards all DOM extracts or fractions. However, PW_bulk_ moderately inhibited the human dermatophyte *Trichophyton* *rubrum*, causative agent of nail and skin infections, including athlete’s foot, with an IC_50_ value of 18.2 µg/mL (Table 1).

As for the bioactivity against dermatological/cosmetical panel, none of the DOM samples exhibited activity against the acne-causing bacterium *Cutibacterium* *acnes* and they were devoid of any inhibitory activity against tyrosinase enzyme. However, the majority of the samples displayed activity against *Staphylococcus* *epidermidis,* a gram-positive biofilm forming bacterium that can cause acne and implant infections (Table 1). Four samples inhibited *S.* *epidermidis* with IC_50_ values lower than that of the positive control drug chloramphenicol (IC_50_ value 8.0 µg/mL), this included P_UW_ (IC_50_ 4.0 µg/mL), P_MeOH_ (IC_50_ 7.2 µg/mL), FW_bulk_ (IC_50_ 6.8 µg/mL) and FW_80MeOH_ (IC_50_ 2.2 µg/mL). Notably, the latter DOM fraction had 4 times higher potency against *S.* *epidermidis* than the control drug*.*

In our study, antibacterial activities are related to high aromaticity content (see the aromatic molecular category and AImod_w_, Table 2). This is also reflected in the PCoA plot (Fig. 3), where the same parameters best explain the variability of the two sample clusters (i.e., peat and freshwater) with highest antibacterial activities. In fact, a significant positive linear regression of *S.* *epidermis* vs X-axis PCoA score has been found (R^2^ = 0.66**) (Additional file 1: Fig. S1a). In addition, significant positive linear regressions with *S.* *epidermis* were also obtained for the same strain against Un_O-rich_ (R^2^ = 0.45*) and Un_with N_ (R^2^ = 0.38*) (Additional file 1: Fig. S1b, c). These positive trends can be explained by a higher content of these molecular category in P samples, the ones with highest antibacterial potential against *S.* *epidermis*. However, PW samples were also abundant in unsaturated molecular formulae and did not show the highest inhibitions of *S.* *epidermis*. The molecular variability in PW was highly explained by H-un molecular formulae (Fig. 3). In this DOM source, two of them did show antibacterial activity (Table 1). Unfortunately, insufficient amounts of sample for the bioactivity tests did not allow us to elucidate the antibacterial potential of all extracts. In *S.* *aureus*, only a significant negative relationship was found with H.Un_O-rich_ (R^2^ = 0.49*) (Additional file 1: Fig. S1d).

**Table 2:** Molecular composition of the different DOM sources and their fractions

Samples	Number of formulas	% of exclusive formulas	MW_w_	DBE_w_	AImod_w_	Aromatic		Highly unsaturated		Unsaturated			Saturated	
						O-rich	O-poor	O-rich	O-poor	O-rich	O-poor	With N	O-rich	O-poor
P_NaOH_	3785	1.1	477	14.7	0.5	10.9	37.5	10.8	36.2	0.2	4.4	0.0	0.0	0.0
P_MQ_	3642	0.3	432	12.5	0.4	8.1	32.0	15.4	37.9	2.0	4.6	0.1	0.0	0.1
P_MeOH_	6465	2.7	530	12.6	0.3	3.8	21.7	5.4	44.2	0.9	23.4	2.0	0.3	0.5
P_EA_	5825	5.9	565	11.5	0.3	1.2	14.2	1.7	50.4	0.6	31.7	0.6	0.1	0.1
PW_pool_	4777	0.3	385	8.5	0.3	3.8	11.7	24.7	42.5	4.4	12.8	3.4	0.0	0.1
PW_50MeOH_	5765	0.5	401	8.4	0.2	4.8	12.0	36.2	21.8	20.3	4.5	13.9	0.3	0.0
PW_80MeOH_	6043	0.2	422	9.9	0.3	4.1	13.2	30.5	40.9	3.3	7.8	2.8	0.0	0.1
PW_100EA_	6377	0.4	430	10.3	0.3	2.2	15.9	12.0	58.9	0.9	10.0	1.5	0.1	0.0
DW_pool_	3032	0.1	474	9.5	0.2	0.1	3.3	27.7	57.9	4.6	5.9	2.8	0.5	0.0
DW_50MeOH_	3379	0.3	437	9.3	0.2	0.1	7.5	36.0	41.6	10.9	3.7	6.6	0.0	0.1
DW_80MeOH_	3231	0.2	496	10.2	0.2	0.1	2.8	30.2	60.4	2.4	4.2	1.1	0.1	0.0
DW_100EA_	2762	0.1	578	11.2	0.2	0.0	1.9	17.4	71.4	3.1	5.8	2.5	0.5	0.0
FW_pool_	4315	0.3	445	11.9	0.4	11.0	20.2	36.7	29.7	0.8	1.6	0.0	0.0	0.0
FW_50MeOH_	3636	0.3	408	11.1	0.4	11.2	28.4	42.7	18.2	1.8	1.2	0.1	0.0	0.0
FW_80MeOH_	2656	0.0	422	11.4	0.4	7.7	23.1	24.6	41.5	0.6	2.6	0.0	0.0	0.0
FW_100EA_	4652	0.2	536	13.1	0.4	3.9	18.2	6.6	61.2	6.0	3.3	0.9	0.7	0.0

P (Peat), FW (Freshwater), PW (Porewater), DW (Deep water); the values of the molecular categories represent the percentage of the total number of molecular formulas

Because of the excessive molecular diversity of the DOM polarity fraction, we performed a holistic molecular characterization on a molecular formula level. In order to identify potential candidates of individual bioactive compounds, we identified those molecular formulae with the highest intensities of the most bioactive samples, and searched for those in public data bases of compounds. We emphasize that this approach yields potential candidates but no unambiguous compound identification. In high-throughput screenings for the current drug discovery pipeline, in which large libraries of compounds are screened against the target of interest, the hit rates in typically less than 1% in most assays [37]. With the advent of machine learning-based screening strategies, efficiencies are greatly enhanced [38]. The results from this study together with the machine-learning implementation techniques should be considered as potential and motivating follow-up studies.

Counting only the formulae with the highest intensities of the most bioactive samples (i.e., 25% of the total intensity), more than 30% of these formulae fall into the category of aromatics (data not shown). A deeper look at the five most intense molecular formulae from the samples with highest activities has been conducted. These will also be applied to the antifungal and antioxidant activity sections. We picked the five most intense molecular formulae (C_13_H_8_O_6_, C_14_H_10_O_6_, C_15_H_12_O_7_, C_15_H_12_O_6_, C_14_H_10_O_5_) from the samples with highest activities against *S.* *aureus* and *S.* *epidermis* and searched data bases for known biochemicals with thes same molecular formulae. Three annotations for the molecular formula C_13_H_8_O_6_ with antibacterial activity have been found: (1) lamellicolic anhydride, a structurally unique aromatic polyketide named phenalenone, reported from the marine-derived fungus *Coniothyrium* *cereale* [39, 40] and from the fungus *Verticillium* *lamellicola* [41, 42]. *Coniothyrium* *cereale* was isolated from the green alga *Enteromorpha* *sp.* This fast-growing and opportunistic macroalgae are present in the environments of this study that show high bioactivity, namely Suwannee River [43] and in sediments of the Wadden Sea [44]. Nevertheless, no records of this algae have been found in Vehnemoor. Albeit phenaloenones have showed previous antimicrobial, anticancer and cytotoxic activities, no bioactivities had been reported for lamellicolic anhydride to date [45]; (2) cladophorol A, isolated from the green alga *Cladophora* *socialis* and showed antibacterial activity against *Plasmodium* *falciparum* with an EC_50_ value of 0.7 µg/mL [46]. The majority *Cladophora* species are distributed throughout the world, are very common and occur almost everywhere: in lakes, dam reservoirs, large rivers and in the coastal littoral zone [47]. In C_14_H_10_O_6_, antibacterial activitiy was reported for juglomicin A, isolated from *Streptomyces* sp., against Gram-positive bacteria such as *Bacillus* *subtilis*, *Staphylococcus* *aureus*, and *Streptococcus* *pneumoniae*, and Gram-negative bacteria such as *Escherichia* *coli*, and *Mycobacterium* *tuberculosis* [48, 49]. *Streptomyces* sp. includes more than 500 species [50] and are widely distributed in soils, exceeding in abundance the other soil bacterial genera [51]. Antibacterial potential was subsequently confirmed, with minimum inhibitory concentrations (MIC) of 6.8, 3.4 and 6.8 µg/mL for *Escherichia* *coli*, *Bacillus* *thuringiensis* and *Xanthobacter* *flavus*, respectively [52]. Taxifolin (C_15_H_12_O_7_), isolated from the mangrove derived actinobacterium *Streptomyces* *sampsonii* (PM33), was found to be active against biofouling bacteria [53]. Another study isolated a compound, named 7-hydroxy-6-methoxy-4-oxo-3-[(1E)-3-oxobut-1-en-1-yl]-4H-chromen-5-carboxylic acid, with this molecular formula from marine-derived fungi [54] that was capable of impairing the biofilm forming ability of *Escherichia* *coli* ATCC 25922. C_15_H_12_O_6_ was also present substantially in samples with the highest antibacterial activity. This natural product, identified as violaceic acid or funalenone, has been detected in *Pseudoalteromonas* and *Vibrionaceae* [55]. However, no potential candidates with specific antimicrobial properties have been found [55]. C_14_H_10_O_5_ was related to two xantones and were identified from the marine algal-derived endophytic fungus *Talaromyces* *islandicus* EN-501 [56]. The genus *Talaromyces* is widely distributed in soil, plants, sponges, foods and in marine environments [57]. Apart from their potent antioxidant activity, these compounds showed antibacterial activity against three human pathogens (*Escherichia* *coli*, *Pseudomonas* *aeruginosa*, and *Staphylococcus* *aureus*) and three aquatic bacteria (*Vibrio* *alginolyticus*, *V.* *harveyi*, and *V.* *parahaemolyticus*), with MIC values ranging from 4 to 32 μg/mL [56].

Interestingly, for the bacterial strain *Enterococcus* *casseliflavus*, activity was only found in the fraction DW_100EA_ and not for the pool from which it originates (i.e., DW_pool_). This sample is highly dominated by highly-unsaturated (O-poor) molecular formulae (71.4%; Table 2). In addition, the PCoA shows how this molecular category strongly explains the molecular composition of this sample. Furthermore, this was the only fraction that showed a slight antitumour activity for all three cell types tested (Table 1). Upon examining the formulae exclusively contained within this fraction, the majority of them belong to the highly-unsaturated (O-poor) category. Interestingly, the molecular weights were at the upper measurement limit, ranging from 856 to 995 Da. Having a deeper look at the five most intense molecular formulae, namely C_47_H_66_O_18_, C_50_H_70_O_20_, C_49_H_68_O_19_, C_48_H_68_O_20_ and C_51_H_70_O_19_, no hints of antibacterial potential were found to date. In case of longer molecules, it is expected that the chance of finding any hits falls off dramatically [58].

Invasive antifungal infections are a severe hazard to human health and the outcome from antifungal treatments is still far from satisfactory [59, 60]. Compared to those available to treat bacterial infections, the number of therapeutic choices for invasive fungal infections is more limited [61]. In this study, no activity against the yeasts *Candida* *albicans* and *Cryptococcus* *neoformans* was observed, whereas PW_bulk_ showed a moderate but specific bioactivity against the pathogenic fungus *Trichophyton* *rubrum*. This sample was abundant in highly-unsaturated (O-rich) and unsaturated molecules (O-poor and with N) (Table 1; Fig. 3). However, upon examining the formulae exclusively contained within this fraction, unsaturated formulae with S were the most dominant. Among the five most intense ones, C_21_H_43_O_9_P, associated to bacilysocin, was found to have activity against certain fungi [62, 63]. This glycerophosphoglycerol was previously isolated from *Bacillus* *subtillis*, a gram-positive bacterium commonly found in soils, rivers and estuarine waters being able to survive for extended periods under adverse environmental conditions [64]. For the other four molecular formulae with highest intensities, C_20_H_41_O_9_P, C_23_H_44_O_11_S, C_22_H_20_N_4_O_9_ and C_24_H_24_N_4_O_9_, no antifungal activity annotations have been found in the literature.

#### Anticancer activity

No significant cytotoxicity was observed against human cancer cell lines, i.e., malignant melanoma (A-375), colon cancer (HCT-116) or breast cancer (MB-231) at the initial test concentration (200 µg/mL). Marginal inhibitory activity (14–17% inhibition at 200 µg/mL) was exhibited by DW_100EA_ (Table 1). Also DW_bulk_ exerted a very low (23%) inhibitory potential against human breast cancer MB-231 (data not shown). However, these activities are too low to determine IC_50_ values, and can be disregarded.

#### Antioxidant / radical scavenging activity

Overall, low levels of antioxidant activity were observed (Table 1). In the first cell-free assay (DPPH), free radical scavenging activity was displayed by 5 samples, with P_MeOH_ being the most active (IC_50_ 61.7 µg/mL). The remaining extracts had weak activities (IC_50_ values ranging from 135.7 to 195.0 µg/mL). In the cell-based antioxidant assay (CAA), only the P_MeOH_ and DW_100EA_ had activity, i.e., 53% and 51% inhibition at the initial 200 µg/mL concentration. However due to low sample amounts, we could not determine their IC_50_ values.

In a previous study, the antioxidant potential of DOM was linked to polyphenols, unsaturated formulae and S-containing compounds, together with high aromaticities [34]. In this study, the best antioxidant activity was exhibited by P_MeOH_ in both DPPH and CAA assays (Table 1). Except for PW_80MeOH_, samples with radical scavenging activity are highly influenced by high DBE, AI.mod, and A_O-poor_ compounds (Fig. 3). These findings are expected since antioxidants should contain double bonds and little oxygen to be reactive [65, 66]. In fact, a significant positive relationship was found in antioxidant activity vs DBE (R^2^ = 0.56*) (Additional file 1: Fig. S1e). Polyphenolic compounds are among the interesting antioxidant compounds isolated from marine sources [67], even though they are originally considered one of the most numerous and ubiquitous groups of substances in the plant kingdom [68], associated with condensed tannins or flavonoids [69] or lignin-like compounds [70]. Although polyphenols have not been included as a main category in this work as they overlap with the category of aromatics, we have also found a positive relationship of polyphenols with antioxidant activity (R^2^ = 0.54*) (Additional file 1: Fig. S1f).

Unsaturated formulae could also play a significant role in the antioxidant potential, given its high presence in P and PW samples (Table 2), as well as its strong influence to explain the molecular composition in PW_80MeOH_ (Fig. 3). We initially thought that S-containing compounds are substantially influencing the high antioxidant potential found in PW_80MeOH_, like in [34]. However, only 8% of the formulae that represent 25% of the total intensity contain sulfur and 80% of them falls in the highly-unsaturated category. This means that highly-unsaturated formulae are probably also playing an antioxidant activity role. In fact, this molecular category is also present in samples with highest antioxidant activities, with values ranging from 53% in P_EA_ to 90% in FW_bulk_. Nor do any of the five most intense formulae, namely C_14_H_16_O_6_, C_13_H_18_O_6_, C_14_H_20_O_6_, C_14_H_18_O_5_ and C_13_H_16_O_7_, contained sulfur. No antioxidant activity annotations have been found in the literature in any of these molecular formulae.

For comparative reasons, antioxidant activity values were normalized by dry weight of DOM and % of radical scavenging was converted in TEAC units by using our own Trolox standard curve (R^2^ = 0.99, p < 0.001) [34]. In this study, antioxidant activities at IC_50_ were around 120 µmol TEAC g^−1^ DW DOM. These values resemble the antioxidant values from water column DOM [34] and those from marshes and rivers [33]. Antioxidant values of peat samples included in this study were higher than other peat extracts, which values ranged between 22 and 57 µmol TEAC g^−1^ DW [71]. This study does not show the porewater samples as the ones with maximum values, which were approximately ten times higher than those from the water column in [34]. This could be because all samples were measured at the same initial concentration and because terrestrial samples were included in this study. Although the methods applied were different (ABTS and DPPH), they should not significantly bias the results obtained, given a significant and high proved correlation between both methods (R^2^ = 0.9) [35].

### Molecular composition

**Fig. 1:**
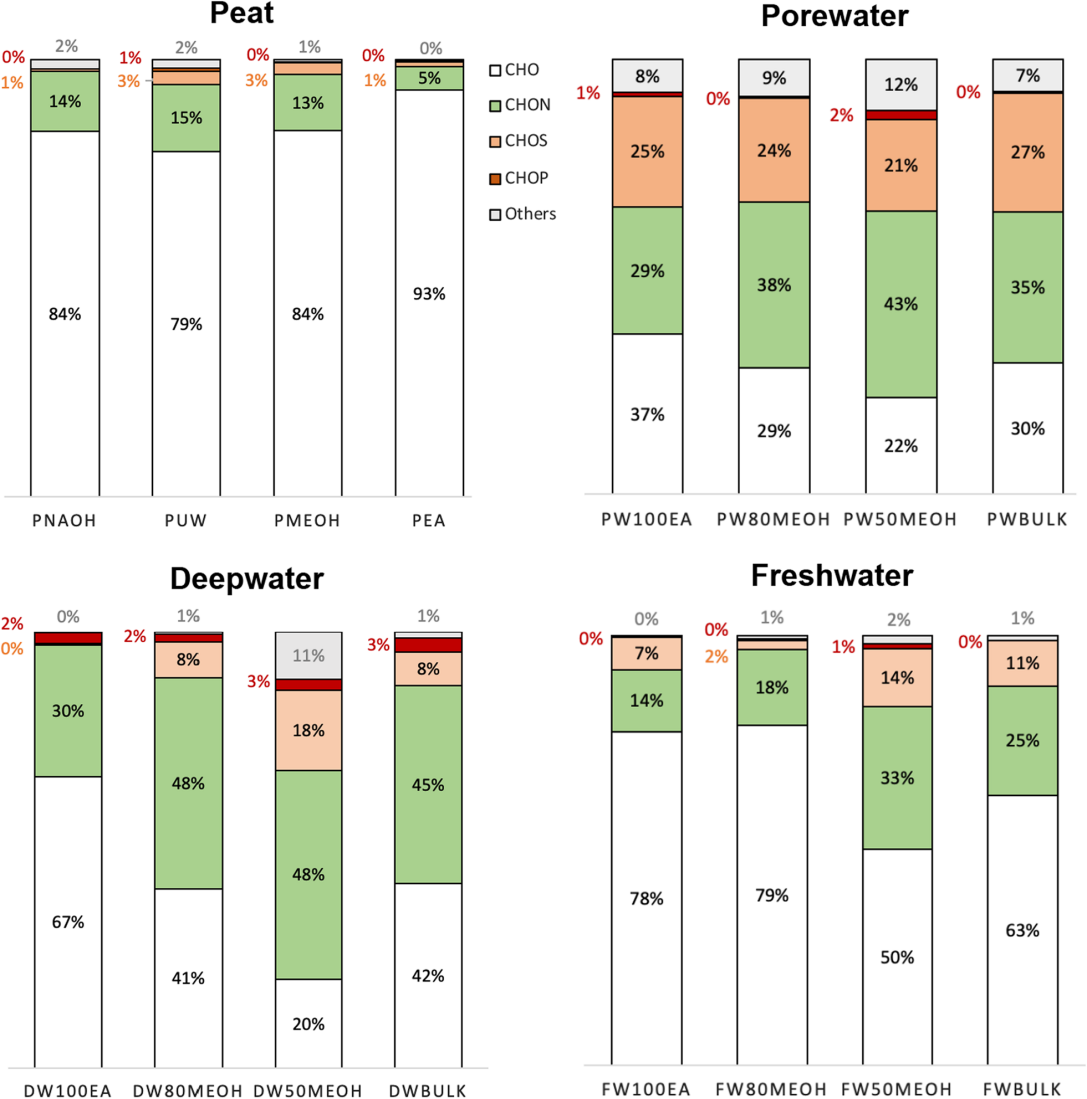
Heteroatomic composition in relative abundance of the different SPEDOM and fractions. The colours denote different atomic compositions: white (CHO), green (CHON), salmon (CHOS), red (CHOP), and gray (Others). The term “Others” refers to the formulas with CHONS, CHOSP, and CHONP

**Fig. 2:**
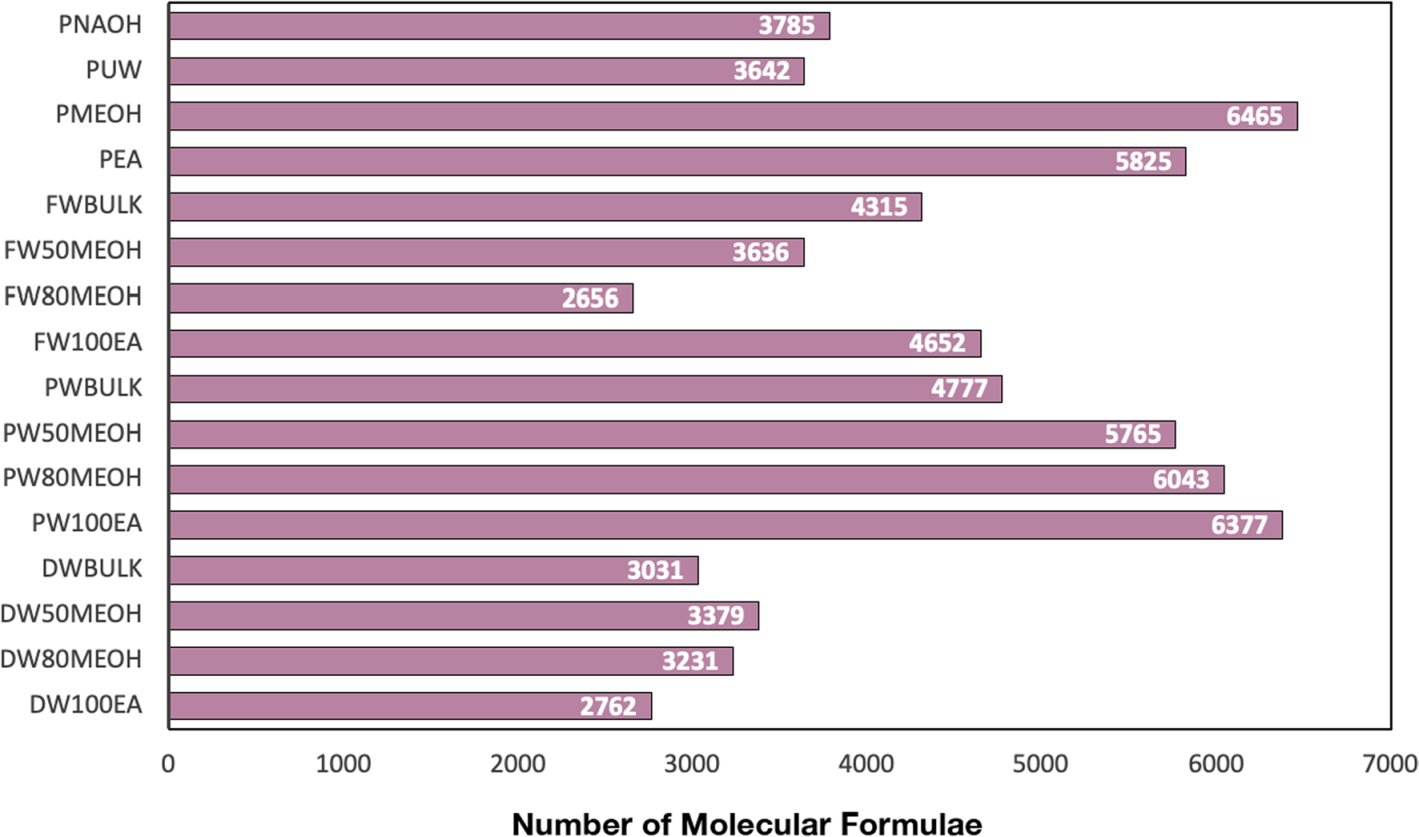
Number of molecular formulae of the different SPE-DOM and fractions

An in-depth non-targeted molecular characterization via FT-ICR MS was done, with the final aim to obtain a broad compositional overview on a molecular formula level, and to link it to observed biological activities. Differences in the molecular composition among the different DOM sources and fractions were observed in the contribution of molecular categories (Table 2), heteroatomic composition (Fig. 1) and number of molecular formulae (Fig. 2). The SPE method chosen here is among the most standardized methods for DOM extraction. In our study, the recoveries of bulk DOM were within the range of what is commonly found in porewater [72–74] and the marine water column [75–77]. In the water column, the extraction efficiency was 61% [78], whereas in samples with more terrestrial influence, extraction efficiencies were relatively lower, namely 31% and 49% and 61% for P_NaOH_ and PW, respectively. In sum, fractionation yields, defined as the DOM recovery eluted in a fraction referred to the initial amount, of 52%, 76% and 89% were achieved in PW, DW and FW fractions, respectively (Additional file 1: Table S1).

In this work, specific molecular formulae of DOM from peat, freshwater, deep water and porewater were presented. Out of the 20,378 molecular formulae, 9035 were present in P, 7017 in DW, 11,830 in PW and 7902 in FW. The highest number of exclusive formulae (formulae only present in a specific sample) was found in P_100EA_ with 5.9% of the dataset, whereas samples such as FW_80MeOH_ contained no exclusive formulae (Table 2).

P samples showed the highest CHO proportions (Fig. 1). P_NaOH_ showed the highest DBE_w_ and A_O-poor_ values with 14.7 and 37.5%, respectively (Table 2). In P_MeOH_ and P_EA_, the maximum Un_O-poor_ values were found at 23.4 and 31.7, respectively.

FW samples, together with P samples, are dominant in CHO formulae. Except FW_50MeOH_, all FW samples exceed 60% of CHO formulae. FW_pool_ and FW_50MeOH_ showed the highest H-Un_O-rich_ values (Table 2). FW_100EA_ showed high values of DBE_w_, H-Un_O-poor_ and S_O-rich_.

PW was the one that presented the proportions more equitably, highlighting the high values of CHOS compared to the rest of the DOM origins. PW also showed the highest number of formulae, exceeding all fractions what was found in the original pool (Table 2, Fig. 2). The maximum number of formulae was 6377 in PW_100EA_ (Table 2, Fig. 2). Fraction PW_50MeOH_ showed the highest percentage of Un_O-rich_ and Un_withN_ (i.e. 20.3% and 13.9%) (Table 2).

DW is more enriched in CHON formulae than the rest of DOM origins, with a maximum of 48% in DW_50MeOH_ (Fig. 1). DW samples also showed the highest proportion of H-Un_O-poor_. The maximum was found in DW_100EA_ at 71.4%. The same sample presented a high value of S_O-rich_ (i.e., 0.5%) (Table 2).

**Fig. 3:**
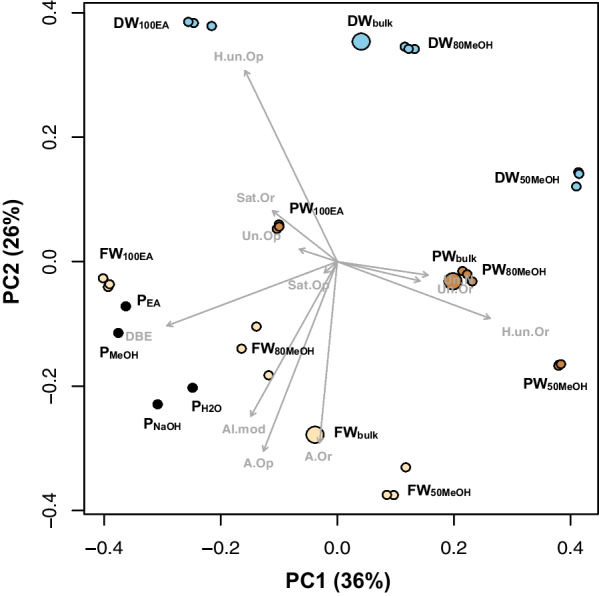
Principal coordinate analysis (PCoA) of the relative abundance of DOM molecular formulas in the whole dataset. Coloured circles refers to the three DOM origins namely peat (P) (black), freshwater (FW) (yellow), porewater (PW) (brown), deep water (DW) (blue), with bigger and smaller circles representing the bulk SPE-DOM and fractions, respectively. 50MeOH (methanol:water (50:50)); 80MeOH (methanol:water (80:20)); 100EA (100% ethyl acetate); H. Un. (highly unsaturated); Un (unsaturated); A (aromatic); Sat (saturated); Or (oxygen rich); Op (oxygen poor); DBE (double bonds equivalent); AI.mod (modified aromatic index)

The PCoA analysis, which separates the samples according to the molecular composition, set the samples in three apparent groups: DW on the top right side (blue colour), P and FW on the bottom left corner (black and light yellow colours), and PW on the bottom right corner (brown colour) (Fig. 3). The samples were correlated to all molecular categories (p < 0.05). In the ordination plots, the first two axes (PC1 and PC2) explained 62% of the DOM molecular variability. DW is clearly more influenced by PC2 and it is separated from the other clusters substantially, indicating a more dissimilar composition from the rest (Fig. 3). In PCoA, the projections of the vectors onto the sampling points depict correlations with the corresponding molecular categories. The aromatic arrows and H-un_O-poor_ define the Y axis, whereas DBE and H-un_O-rich_ define the X axis. For instance, the DW_100EA_ fraction correlated strongly with the molecular category H-un_O-poor_, PW_100EA_ correlated with Sat_O-rich_ (Fig. 3). H-un_O-rich_ correlated strongly with PW_50MeOH_. Un_O-rich_ and Un_N-rich_ correlated with PW and PW_80MeOH_. Terrestrial samples (FW and P) correlated with A_O-poor_ and A_O-rich_, as well as with AI_mod_ (Fig. 3). DBE correlated mostly with P samples.

It is important to emphasise that fractions still remain very complicated at the molecular level, making the full structural elucidation unfeasible even after fractionation. In fact, polarity-mediated fractionation was carried out to achieve separation in the molecular composition and subsequently, in the actibacterial, antifungal, antitumoral and antioxidant potential, rather than to lower the molecular complexity. Our study shows that the molecular composition of P and FW are more similar than the other origins, which also reflects a greater resemblance of bioactivities. Apart from the terrestrial origin of both, our FW sample (i.e., from Suwannee River) was also extracted from peat areas, thus increasing the molecular resemblance.

The relative intensity of each formula is a semi-quantitative proxy for its contribution in a sample. On this regard, in the next sub-sections, we deploy the most intense molecular formulae for those extracts with the highest bioactivities and the search for possible candidates with bioactive potential.

## Conclusions

This study demonstrated the potential of bulk DOM and its fractions from various marine and terrestrial sources for pharmaceutical and cosmeceutical applications. To the best of our knowledge, this is the first broad spectrum screening study showing the antibacterial and antifungal activities of DOM, together with the already known antioxidant potential. No cytotoxic activity was observed, which points out a general safety of the DOM extracts. Furthermore, the molecular characterization of DOM using ultra-high resolution mass spectrometry allowed for a deeper understanding of the DOM composition and the prediction of possible molecular formulae responsible of the bioactivity. Several previously unknown candidates of bioactive compounds have been tentatively identified, which illustrates the potential of DOM to future studies to focus on these molecular formulae. Many of them originate from microorganisms with a wide distribution, including the environments sampled in this study, so these potential compounds could probabilistically be derived from these organisms.

Anthropogenic pharmaceuticals, a major group of emerging pollutants, may possibly contribute to the bioactivity of the DOM samples considered in our study. Metagenomic profiles of antibiotic resistance genes in coastal [79] and even hadal sediments [80] indicate the presence of anthropogenic antibiotics not only in regions of immediate human influence but also in some of the most remote parts of the ocean floor. However, none of the molecular formulae that corresponded to the most commonly used antimicrobials found in the marine environment [81] were found in our study. This is not surprising because the deep Pacific Water considered in our study is one of the oldest water masses on Earth and has not been in contact with the atmosphere for several centuries [82], while the other samples are from biologically very productive regions and not exposed to direct pharmaceutical efflux. We encourage future studies to investigate further whether trace amounts of pharmaceutical pollutants in natural waters may exhibit detectable bioactivity against the large background of natural DOM.

Contrary to conventional biodiscovery studies, here we worked on inseparable mixtures that are not fully characterized on a molecular structural level. Since the chemical complexity and low supply of DOM material through laboratory scale extractions currently hampers isolation of the individual DOM constituents, new technical and methodological improvements are needed to address full pharmaceutical potential of DOM. Although bulk DOM extracts or the polarity fractions may be useful for the cosmetic industry, pharmaceutical applications require highly purified, fully chemically characterized compounds with potent activity. The major challenge in this respect is the isolation of single DOM compounds that are responsible for a particular bioactivity in sufficient quantities. Modern screening technologies, such as multiplexed high-throughput molecular-phenotypic screening, combined with untargeted metabolomics and multivariate statistical analyses, are providing first insights on previously undetectable DOM bioactivities [83]. Major efforts are needed for an industrial scale DOM extraction, coupled with a novel systematic combinatorial workflow for bioactivity testing and by compound purification, together with the characterization by high resolution analytical and spectroscopic techniques to afford the individual bioactive components in sufficient yields.

## Experimental section

### DOM collection and extraction

In this first bioactivity screening, DOM samples were sourced from different environments to cover a wide spectrum. Peat sample (P) was collected in Vehnemoor in Lower Saxony, Germany (53° 06ʹ 58ʺ N, 7° 98ʹ 19ʺ E). Today, Vehnemoor is dominated by moist to wet, locally fresh, mostly drained, nutrient-poor raised bog soils [84]. Despite having been affected by the widespread peat extraction until 2020, this area shows emblematic features closer to its former natural character full of wetland habitats all year around [84]. The sample was ground and extracted by different solvents separately starting with the same peat source material, namely ultrapure water (P_UW_, ultrapure water obtained by Sartorius equipment, at a concentration of 40 g/L), methanol (P_MeOH_, UPLC/MS grade, Sigma-Aldrich, at a concentration of 58 g/L), ethyl acetate (P_EA_, HPLC grade, VWR Chemicals, at a concentration of 58 g/L) and sodium hydroxide (P_NaOH_, > 32% pure, Carl Roth, at a concentration of 29 g/L) (NaOH at 0.01 M (pH = 12)). Samples were stired at room temperature for 24 h. Afterwards, the different peat extracts were filtered through 0.45 μm Whatman GF/F precombusted (450◦ C, 4 h) filters in an acid-cleaned filtration system. To remove the NaOH salt, the NaOH extract (P_NaOH_) was solid phase extracted (SPE) after adjusting the pH to 2.0 with HCl (25%, p.a.) by using 5-g PPL cartridges (Agilent), formerly rinsed with MeOH (UPLC/MS grade, Biosolve BV). Before DOM elution, the cartridges were rinsed several times with ultrapure water, acidified at pH 2 with HCl (25%, p.a.) to remove the salt from the cartridges (a prerequisite for MS analysis), and then dried under a stream of ultrapure N_2_. Elution of the SPE-DOM from the PPL columns was performed with 40 ml of MeOH. The final SPE-extract was stored in MeOH at -20 °C at a concentration of 7 mmol/L.

The deep water (DW) sample was collected at the Natural Energy Laboratory of Hawaii Authority (NELHA; www.nelha.org) on the island of Big Island (19° 44′ N, 156° 04′ W), Hawaii, United States, by the Research Group for Marine Geochemistry (ICBM, Oldenburg) in 2008 [78]. At NELHA, the North Equatorial Pacific Intermediate Water (674 m; NEqPIW), one of the oldest water masses on Earth, was accessed. It was filtered directly at the NELHA laboratory taps through a 0.2 μm filter (Causa-PES 0.2 μm polyether sulfone final filters for PPL, as described in [78]. The final SPE-extract, namely DW_bulk_, was stored in methanol at − 20 °C at a concentration of 750 mmol/L.

Thirdly, sulfidic sediment porewater (PW) from the extensive tidal flats of the Wadden Sea close to the Island of Spiekeroog (Germany) was also collected (see [85] for more information). The porewater was collected in 10 L acid-cleaned polycarbonate carboys by digging a hole directly in the intertidal flatland, transported directly to the laboratory and stored in the dark at 4 °C until filtration within 24 h. Filtration was performed first through acid-cleaned 1 μm GMF filters (Whatman) and then through pre-combusted (450 °C, 4 h) 0.7 μm Whatman GF/F filters in an acid-cleaned filtration system. Filtered water was acidified to pH 2 (25% HCl, p.a.). Desalination of the samples and DOM extraction was done via SPE using 5-g PPL cartridges (Agilent) by the same protocol described above with P_NaOH_. The final SPE-extract, PW_bulk_, was stored in methanol at − 20 °C at a concentration of 25 mmol/L.

Fourthly, a freshwater DOM sample (FW) from Suwannee River (SRNOM; International Humic Substances Society (IHSS), St Paul, MN, United States), hereafter FW_bulk_, was purchased directly from the IHSS. This sample was extracted via reverse osmosis system (RO) [86].

Non-purgeable dissolved organic carbon (DOC) and total dissolved nitrogen (TDN) were measured on a Shimadzu TOC-VCPH and TNM-1 nitrogen detectors. DOC refers specifically to the mass of carbon in the dissolved organic material and there is typically about twice as much DOM as DOC [87].

DOC and TDN were determined by drying an aliquot of the extract at 36 °C and redissolved with ultrapure water at pH 2. Routine minimum detection limits are 10 μM-C for DOC and 6 μM-N for TDN, and standard errors are typically < 2.5% of the DOC or TDN concentrations [88]. Deep sea reference samples provided by D. Hansell (University of Miami, United States) were included in the analysis for validation.

### DOM fractionation

DW_bulk_, PW_bulk_ and FW_bulk_ DOM were further polarity fractionated by SPE using 5 g size PPL cartridges (Agilent). 25 mg of each extract were passed via gravity through a 5 g PPL cartridge after adjusting the pH of the sample solution to pH 2 (25% HCl, p.a.). Afterwards, extracts were sequentially eluted with different solvent mixtures following a polar to apolar gradient. Fractionation yields for each fraction were calculated, according to the amount of carbon recovered (Additional file 1: Table S1). All organic solvents were MS grade (see further details in Sect. 2.1) and water had ultrapure quality (obtained from Arium Pro DI Ultrapure Water System). For fractionation, mixtures of methanol:water (ratio 50:50, i.e., subscript _50MeOH_), methanol:water (80:20, i.e., subscript _80MeOH_) and ethyl acetate (100%, i.e., subscript _100EA_) were used. The fractions were stored at − 20 °C, and prior to further measurements, they were dried and re-dissolved in the corresponding solvent mixture.

### Biological activity tests

DOM extracts and their fractions with sufficient supply were subjected to six panels of bioassays. This included (i) clinically relevant human pathogenic bacteria *Staphylococcus* *aureus*, *Pseudomonas* *aeruginosa*, *Enterococcus* *faecium*, *Enterococcus* *faecalis*, *Enterococcus* *casseliflavus*, (ii) Fish/shellfish pathogens *Lactococcus* *garvieae* and *Vibrio* *parahaemolyticus* that are not only relevant for aquaculture, but also transmitted to human by seafood consumption (iii) human pathogenic yeasts/fungi *Candida* *albicans*, *Cryptococcus* *neoformans* and *Trichophyton* *rubrum,* (iv) human cancer cell lines, melanoma (A-375), colon cancer (HCT-116) and breast cancer (MDA-MB-231), (v) assays relevant for cosmetics/dermatological applications, i.e., *Cutibacterium* *acnes*, *Staphylococcus* *epidermidis*, and tyrosinase enzyme inhibitory activity, (vi) antioxidant potential via cell-free DPPH assay and the cellular antioxidant assay (CAA). All bioassays were performed in duplicates using 96-well microplates at an initial test concentration of 200 µg/mL. The samples that showed inhibitory rate of ≥ 50% at this concentration were submitted to IC_50_ determinations (when the sample amounts were sufficient). For this, a dilution series was prepared and the IC_50_ value was calculated as the concentration that show 50% inhibition of viability based on a negative control (DMSO). More details on assays against pathogenic microorganisms are shown in Additional file 1: Table S2.

#### Bacterial and yeast assays

All test organisms were purchased from Leibniz Institute DSMZ (Braunschweig, Germany). *Staphylococcus* *aureus* DSM 346, *Staphylococcus* *epidermidis* DSM 20044, *Pseudomonas* *aeruginosa* DSM 1128 and *Vibrio* *parahaemolyticus* DSM 11058 were cultivated in TSB12 medium (1.2% tryptic soy broth, 0.5% NaCl), *Enterococcus* *faecium* DSM 20477*,* *Enterococcus* *faecalis* DSM 20478, *Enterococcus* *casseliflavus* DSM 7370 and *Lactococcus* *garvieae* DSM 20684 in Medium 92 (as described on DSMZ webpage www.dsmz.de), *Cutibacterium* *acnes* DSM 1897 in Medium 104 (as described on DSMZ webpage www.dsmz.de), *Candida* *albicans* DSM 1386 in Medium 186/3 (0.33% glucose, 0.17% peptone from soybeans, 0.1% yeast extract, 0.1% malt extract) and *Cryptococcus* *neoformans* in Medium 186 (as described on DSMZ webpage www.dsmz.de).

Test strains were incubated overnight in their respective medium, except *C.* *acnes* for 48 h, and diluted to an optical density (600 nm) of 0.01–0.03. The test samples (40 mg/mL DMSO stock solution) were dissolved in medium and transferred into a 96-well microtiter plate and 200 µl of the cell suspension cultures were added to each well. Microplates were incubated for 5–18 h at 22–37 °C and shaken at 200 rpm whenever necessary (see Additional file 1: Table S2). *C.* *acnes* was cultivated for 48 h at 37 °C in a closed chamber flushed with nitrogen for 10 min. Subsequently, 10 µL resazurin solution (0.3 mg/ml in phosphate buffer) was added to each well and the microplates were incubated again for 5–60 min before measuring fluorescence (560/590 nm) using a microplate reader (Tecan Infinite M200, Tecan, Männedorf, Switzerland). For *Enterococcus* sp., *L.* *garvieae* and *C.* *acnes*, the pH indicator bromocresol purple was used as detection reagent to determine color/pH change (acidification) caused by growth of the respective test strains. Color change was detected by absorbance measurement (600 nm/reference 690 nm). For *C.* *neoformans* the absorbance at 600 nm was measured. The percentage of inhibition was calculated on the basis of a negative control (no extract) and compared to a positive control (standard antibiotic, see Additional file 1: Table S2). The IC_50_ values were calculated as described above.

#### Antifungal assay

Samples were prepared in a microplate and the assay was conducted as previously described [89]. Briefly, to cause sporulation of *Trichophyton* *rubrum* I/95 (patient isolated from University Kiel, Dermatology, Prof. Brasch) was cultivated for two weeks on GPY solid medium (0.1% glucose, 0.05% petone, 0.01 yeast extract, 1.5% agar). A suspension of 5 × 10^4^ spores/ml in liquid Medium 186 was prepared and a volume of 200 µl was added to each microplate well. After incubation of 3 days at 28 °C (see Additional file 1: Table S2), absorbance was measured at 600 nm. The percentage of inhibition and the IC_50_ values were calculated as described above.

#### Anticancer activity

The human malignant melanoma cell line A-375 and breast cancer line MDA-MB-231 was purchased from CLS Cell Lines Service GmbH (Eppelheim, Germany) and the colon cancer cell line HCT-116 from Leibniz Institute DSMZ (Braunschweig, Germany). The antitumoral activity of the test samples was evaluated by monitoring the metabolic activity using the CellTiterBlue Cell Viability Assay (Promega, Mannheim, Germany). A-375 and HCT-116 cells were cultivated in DMEM medium supplemented with 4.5 g/L D-Glucose and 110 mg/L Sodium Pyrovate and MDA-MB-231 cells in DMEM:Ham’s F12 medium (1:1) supplemented with 15 mM HEPES and. All media were supplemented with L-Glutamine, 10% fetal bovine serum, 100 U/mL penicillin and 100 mg/ml streptomycin. The cultures were maintained at 37 °C under a humidified atmosphere and 5% CO_2_. The cell lines were transferred every 3 or 4 d.

For experimental procedure, the cells were seeded in 96 well plates at a concentration of 10,000 cells per well. A stock solution of 40 mg/mL in DMSO was prepared of each extract. After 24 h incubation, the medium was removed from the cells and 100 µl fresh medium containing the test samples was added. Doxorubicin as a standard therapeutic drug was used as positive control, 0.5% DMSO and growth media were used as controls. Following compound addidion, plates were cultured for 24 h at 37 °C. Afterwards, the assay was performed according to the manufacturer’s instructions and measured using the microplate reader Tecan Infinite M200 at excitation 560 nm and emission of 590 nm. The percentage of inhibition was calculated as described above.

#### Tyrosinase enzyme inhibitory activity

Analysis of effects on mushroom tyrosinase was carried out by using the method of [90]. The enzyme was dissolved in 16.7 mM phosphate buffer to a working solution of 200 U/mL. 10 µL of the diluted test samples were added to a clear 96 well microplate and mixed with 90 µl of the enzyme solution. After incubation of 10 min at 25 °C the reaction was started by adding 1.2 mM *L*-tyrosine dissolved in phosphate buffer. The occurred brownish dopychrom was detected by measuring the absorbance at 490 nm after 30 min at 25 °C and the percentage of inhibition was calculated. Kojic acid was used as positive control.

#### DPPH assay

The assay was performed by dissolving the DPPH (2,2-diphenyl-1-picrylhydrazin) in methanol to a final concentration of 200 µM. Samples and the positive control ascorbic acid were prepared in methanol as well. For the test, 100 µL of the sample was pipetted in a clear 96 well microplate and the reaction was started with 100 µL DPPH solution. After 30 min of incubation in the dark at room temperature the antioxidative capability of the samples were measured by photometric appointment at 517 nm using the Tecan Infinite M200. The percentage of inhibition and the IC_50_ values were calculated as described above.

#### CAA assay

Subculturing of cancer cell line A-375 took place as described before. The cellular antioxidant potential of DOM extracts and their fractions was carried out in black 96-well plates with an optical bottom and was performed as previously described [91]. A-375 cells were seeded at a density of approximately 100,000 cells/well and incubated overnight. Cells were then incubated with a final concentration of 25 µM DCFH-DA (2′7′-dichlorofluorescin diacetate) and the test compounds for 1 h. Luteolin was used as positive control. After incubation, Hank’s saline solution without phenol red supplemented with 600 µM AAPH was added to all wells. After 10 min the plate was placed in the plate reader and fluorescence recorded; excitation of 485 nm and emission of 520 nm were used. Cells were washed with Hank’s saline solution between the addition of new reagents. The total reaction volume was 100 μL. The incubations were at 37 °C in a humidified atmosphere of 5% CO_2_. The percentage of inhibition was calculated as described above. Due to lack of sample quantities, we were unable to determine the IC_50_ values of the two active fractions.

### Molecular composition

Small aliquots of all samples were first evaporated to dryness under a stream of nitrogen gas and redissolved in ultrapure water (obtained from Arium Pro DI Ultrapure Water System) and methanol (UPLC/MS grade, Sigma-Aldrich) (1:1, v/v) at DOC concentration of 2.5 mg C L^−1^. Duplicates of each extract were measured using a Solarix XR FT-ICR MS (Bruker Daltonik GmbH) eqquiped with a 15 Tesla superconducting magnet (Bruker Biospin). Samples were injected at a flow rate of 1.5 µL s^−1^ into the electrospray ionization source (ESI; Apollo II ion source, Bruker Daltonik GmbH) and analyzed in negative mode. Ions were accumulated in the hexapole for 0.1 s prior to transfer into the ICR cell. Data acquisition was done in broadband mode with a scanning range of 100–1000 Da and with an accumulation of 200 scans. The calibration of the spectra resulted in a mass error of < 0.1 ppm. Instrument assessment was done with an in-house standard from NELHA station [92, 93]. Method detection limit (MDL), mass alignment of different spectra and molecular formula attribution was done with the software ICBM-OCEAN [94]. The MDL method (MDL level 4) was used to eliminate instrumental noise. Mass spectra were recalibrated to reduce systematic error. Masses considered to be of equal origin were aligned (0.5 ppm tolerance) and averaged over spectra to reduce the random mass error [95]. In the formula attribution, the N, S, P rule and the isotope verification was applied to exclude unlike formulae. In addition to the CH_2_ homologous series, CO_2_, H_2_, H_2_O and O homologous series were considered to improve the formula assignment [95]. Identified contaminants present in spectra were removed prior to statistical analysis. Only the formulae present in both duplicate measurements were considered for further evaluation.

After applying the abovementioned filtration criteria, the number of assigned formulae were 20,378 across all samples. The assigned formulae were sorted into groups of formulae containing the atoms CHO, CHON, CHOS, CHOP, CHONS, CHOSP and CHONP (the latter three are referred to as “others “). In addition, the identified molecular formulae were classified into compound groups based on established molar ratios (H/C, O/C), modified aromatic index (AImod), double bond equivalent (DBE) and heteroatoms contents. The molecular categories correspond to (1) aromatics (A) (AImod ≥ 0.5), (2) highly unsaturated (H-un) (AImod < 0.5, H/C < 1.5), (3) unsaturated (Un) (1.5 ≤ H/C ≤ 2) and (4) saturated (S) (DBE = 0). The four molecular categories were subdivided in oxygen-rich (_O-rich_, O/C > 0.5) and oxygen-poor (_O-poor_, O/C ≤ 0.5), with an extra category for the unsaturated, namely “_with N_” (1.5 ≤ H/C ≤ & N) (Merder et al., 2020). The formulae were normalized to the sum of all molecular formula intensities for each sample, and subsequently, the intensity weighted-averages of elemental ratios H/C and O/C, DBE, AI_mod_ [96, 97] and of the defined molecular categories were calculated. Exclusive molecular formulae present at each sample were also considered. Exclusivity refers to those formulae that are only present in a given sample, but not in the other samples.

### Statistical analysis

Principal coordinate analysis (PCoA) was performed on a Bray Curtis dissimilarity matrix of the normalized peak intensities of all identified DOM molecular formulae as described in [98]. The DOM molecular categories and molecular indexes were fitted post-hoc to the PCoA scores using the envfit function of the vegan package [99] within the R statistical platform [100]. The correlation of molecular parameters to the DOM molecular composition (PCoA) was tested with 10,000 permutations and was considered significant if *p* < 0.1. Linear regressions between molecular parameters and bioactivity data have been calculated only for those bioactivity tests with sufficient data (Additional file 1: Fig. S1).

### Supplementary Information

**Additional file 1: Table S1. **Dissolved organic carbon (DOC) concentrations, DOM extraction efficiencies and fractionation yields**. Table S2. **Bacterial test strains used for assessment of antibacterial activity of DOM extracts and their fractions. **Fig. S1.** Significant Relationships between molecular parameters and bioactivities. **: p < 0.01; *: p < 0.05. *S.* *aureus:* *Staphylococcus* *aureus;* *S.* *epidermis:* *Staphylococcus* *epidermis;* Un: Unsaturated; H. Un: Highly Unsaturated; O-rich: Oxygen rich; O-poor: Oxygen poor; with N: with Nitrogen.

## Data Availability

The data supporting the findings of this study are available upon reasonable request from the corresponding author.
