# Supplementary material for: Bioactivity profile of dissolved organic matter and its relation to molecular composition
Source: Nat Prod Bioprospect. 2023 Sep 18;13(1):32. doi: 10.1007/s13659-023-00395-y (PMC10507005; doi:10.1007/s13659-023-00395-y)
Supplement: Supplementary file 1 — Additional file 1: Table S1. Dissolved organic carbon (DOC) concentrations, DOM extraction efficiencies and fractionation yields. Table S2. Bacterial test strains used for assessment of antibacterial activity of DOM extracts and their fractions. Fig. S1. Significant Relationships between molecular parameters and bioactivities. **: p < 0.01; *: p < 0.05. S. aureus: Staphylococcus aureus; S. epidermis: Staphylococcus epidermis; Un: Unsaturated; H. Un: Highly Unsaturated; O-rich: Oxygen rich; O-poor: Oxygen poor; with N: with Nitrogen. [file 13659_2023_395_MOESM1_ESM.docx]

**Additional file 1**

**Bioactivity Profile of Dissolved Organic Matter and Its Relation to Molecular Composition**

**Teresa S. Catalá^a,b,c*^, Linn G. Speidel^c,d^, Arlette Wenzel-Storjohann^e^, Thorsten Dittmar^c,f^, Deniz Tasdemir^e,g^**

^a^ Global Society Institute, Wälderhaus, Hamburg, Germany

^b^ Organization for Science, Education & Global Society gGmbH, Stuttgart, Germany

^c^ ICBM-MPI Bridging Group for Marine Geochemistry, Institute for Chemistry and Biology of the Marine Environment (ICBM), University of Oldenburg, Oldenburg, Germany

^d^ Geological Institute, Department of Earth Sciences, ETH Zurich, 8092 Zurich, Switzerland

^e^ GEOMAR Centre for Marine Biotechnology, Research Unit Marine Natural Products Chemistry, GEOMAR Helmholtz Centre for Ocean Research Kiel, Am Kiel-Kanal 44, Kiel, 24106, Germany

^f^ Helmholtz Institute for Functional Marine Biodiversity, University of Oldenburg, Oldenburg, Germany

^g^ Kiel University, Christian-Albrechts-Platz 4, Kiel, 24118, Germany

*Corresponding author

Tel: [+49 711 6742 8718](tel:+4971167428718)

E-mail: [teresa.scatala@globalsocietyinstitute.org](mailto:teresa.scatala@globalsocietyinstitute.org)

**Table S1** Dissolved organic carbon (DOC) concentrations, DOM extraction efficiencies and fractionation yields

|  | **Samples** | **DOM Extraction efficiency (%)** | **Fractionation yields (%)** |
| --- | --- | --- | --- |
|  |  |  |  |
| **Peat** | **P_NaOH_** | 31 | - |
|  | **P_UW_** | - | - |
|  | **P_MeOH_** | - | - |
|  | **P_EA_** | - | - |
| **Porewater** | **PW_bulk_** | 49 | - |
|  | **PW_50MeOH_** | - | 53 |
|  | **PW_80MeOH_** | - | 51 |
|  | **PW_100EA_** | - | 52 |
| **Deep Water** | **DW_bulk_** | 61 | - |
|  | **DW_50MeOH_** | - | 73 |
|  | **DW_80MeOH_** | - | 76 |
|  | **DW_100EA_** | - | 79 |
| **Freshwater** | **FW_bulk_** | - | - |
|  | **FW_50MeOH_** | - | 88 |
|  | **FW_80MeOH_** | - | 90 |
|  | **FW_100EA_** | - | 89 |

PW and DW extractions were done by solid phase extraction (SPE) with PPL cartridges (Catalá et al., 2020;

Green et al., 2014). FW was directly acquired from the International Humic Substances Society.

**Table S2:** Bacterial test strains used for assessment of antibacterial activity of DOM extracts and their fractions.

| **Test panel** | **Species name** | **Strain No.** | **medium** | **Pre-culture** | **Microplate growth condidtion** | **Detection reagent** | **positive control** |
| --- | --- | --- | --- | --- | --- | --- | --- |
| clinically relevant human pathogenic bacteria | *Staphylococcus aureus* | DSM 346 | TSB12 | Overnight, 28 °C, 160 rpm | 5 h, 37 °C, 200 rpm | resazurin | Chloramphenicol |
|  | *Pseudomonas aeruginosa* | DSM 1128 | TSB12 | Overnight, 28 °C, 160 rpm | 5 h, 37 °C, 200 rpm | resazurin | Polymyxin B |
|  | *Enterococcus faecium* | DSM 20477 | Medium 92 | Overnight, 37 °C | 5 h, 37°C | bromocresol purple | Ampicillin |
|  | *Enterococcus faecalis* | DSM 20478 | Medium 92 | Overnight, 37 °C | 5 h, 37°C | bromocresol purple | Ampicillin |
|  | *Enterococcus casseliflavus* | DSM 7370 | Medium 92 | Overnight, 37 °C | 7 h, 37 °C, | bromocresol purple | Ampicillin |
| Fish/shellfish pathogens | *Lactococcus garvieae* | DSM 20684 | Medium 92 | Overnight, 37 °C | 5 h, 37°C | bromocresol purple | Ampicillin |
|  | *Vibrio parahaemolyticus* | DSM 11058 | TSB12 | Overnight, 28 °C, 160 rpm | 6 h, 28 °C, 200 rpm | resazurin | Chloramphenicol |
| human pathogenic yeasts/fungi | *Candida albicans* | DSM 1386 | M186/3 | Overnight, 28 °C, 160 rpm | 5 h, 37 °C, 200 rpm | resazurin | Nystatin |
|  | *Cryptococcus neoformans* | DSM 6973 | Medium 186 | Overnight, 28 °C, 160 rpm | 16 h, 28 °C, 200 rpm | - | Amphotericin B |
|  | *Trichophyton rubrum* | I/95 | Medium 186 Agar | 7 d, 28 °C | 72 h, 28 °C, 120 rpm | - | Clotrimazol |
| Cosmetics/dermatolo-gical assays | *Cutibacterium acnes* | DSM 1897 | Medium 104 | 48 h, 37 °C | 48 h, 37 °C, anaerobic | bromocresol purple | Chloramphenicol |
|  | *Staphylococcus epidermidis* | DSM 20044 | TSB12 | Overnight, 28 °C, 160 rpm | 5 h, 37 °C, 200 rpm | resazurin | Chloramphenicol |

**Fig. S1** Significant Relationships between molecular parameters and bioactivities. **: p < 0.01; *: p < 0.05. *S. aureus: Staphylococcus aureus; S. epidermis: Staphylococcus epidermis;* Un: Unsaturated; H. Un: Highly Unsaturated; O-rich: Oxygen rich; O-poor: Oxygen poor; with N: with Nitrogen.
